# Supplementary material for: IgE Occupancy and Antigen Valency Cooperate to Control FcεRI Aggregation Geometry and Signaling Efficiency
Source: Int J Mol Sci. 2026 Jul 28;27(15):6762. doi: 10.3390/ijms27156762 (PMC13466504; doi:10.3390/ijms27156762)
Supplement: Supplementary file 1 [file ijms-27-06762-s001.zip › ijms-4364731 Supplementary Videos_Captions.pdf]

Video S1. Model of a linear aggregate formed by MB1N1C. Linear aggregates consist of alternating antibody-ligand links with no branching. Here, ligand in a linear aggregate is bound to at most two antibodies. Antigen: Gray PDB model of MB1N1C with epitopes identified according to the scheme in Figure 1A. Antibodies: Gray 3D isosurface model of IgE bound to FcεR1α subunit.

Video S2. Model of a linear aggregate formed by MB2N. Linear aggregates consist of alternating antibody-ligand links with no branching. Here, ligand in a linear aggregate is bound to at most two antibodies. Antigen: Gray PDB model of MB2N with epitopes identified according the scheme in Figure 1A. Antibodies: Gray 3D isosurface model of IgE bound to FcεR1α subunit.

Video S3. Model of a complex aggregate formed by MB4N. Complex aggregates consist of alternating antibody-ligand links with branching. Consequently, there exists at least one ligand in a complex aggregate that is bound to at least three antibodies. Antigen: Gray PDB model of MB4N with epitopes identified according to the scheme in Figure 1A. Antibodies: Gray 3D isosurface model of IgE bound to FcεR1α subunit.
