# Supplementary material for: IgE Occupancy and Antigen Valency Cooperate to Control FcεRI Aggregation Geometry and Signaling Efficiency
Source: Int J Mol Sci. 2026 Jul 28;27(15):6762. doi: 10.3390/ijms27156762 (PMC13466504; doi:10.3390/ijms27156762)
Supplement: Supplementary file 1 [file ijms-27-06762-s001.zip › Supplementary Materials_Table S1_Figure S1.pdf]

Supplementary Table S1. Quantification of mIgE-bound FcεRI on RBL-2H3 cells.

mIgE^{Alexa647} concentration for priming of RBL cells (ng/ml)	No. of calculated FcεRI bound to mIgE^{Alexa647} on the cell surface
1200	110577
600	67932
300	54226
150	35045
75	20548
38	14380
19	3795
10	2155

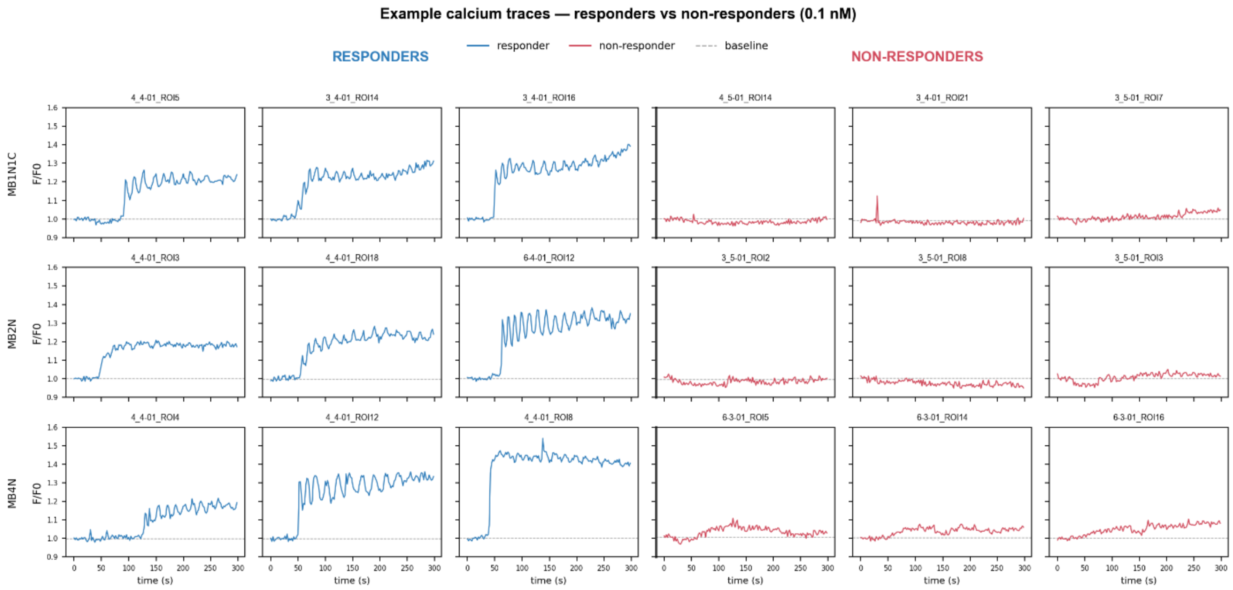

Supplemental Figure S1. Example single cell calcium traces classified as responders and non-responders. Representative Fura-2 ratio traces normalized to the pre-antigen value (F/F0) for each of the three antigen conditions (MB1N1C, MB2N, MB4N) at 0.1 nM concentration: three example responders (blue) and non-responders (red) are shown. Responders were distinguished from non-responders directly from the raw ratio trace. For each trace, a baseline *b* was defined as the median of the pre-antigen frames, and the trace was denoised with a 5-frame moving average. A cell was classified as a *responder* only if it satisfied two criteria: (1) sharp onset, that is the maximum increase of the smoothed trace over any ~20 s sliding window was >= 0.065 ΔF/F; and (2) sustained elevation, where the median of the final 12% of frames exceeded baseline by >= 0.06 ΔF/F. Cells failing either criterion were classified as *non-responders*. Requiring both features identifies a genuine response (a rapid rise to a maintained plateau) while rejecting three artifact classes: slow monotonic drift/creep, single-frame noise spikes, and transient spikes or dip-and-recover excursions. A minority of ambiguous ramp-like cell traces were flagged for manual review and classification was manually decided by trace shape.
